# Development of Statistically Optimized Chemically Cross-Linked Hydrogel for the Sustained-Release Delivery of Favipiravir

**DOI:** 10.3390/polym14122369

**Published:** 2022-06-11

**Authors:** Ahmad Salawi, Arooj Khan, Muhammad Zaman, Tehseen Riaz, Hafsa Ihsan, Muhammad Hammad Butt, Waqar Aman, Rahima Khan, Imtiaz Majeed, Yosif Almoshari, Meshal Alshamrani

**Affiliations:** 1 Department of Pharmaceutics, College of Pharmacy, Jazan University, Jazan 45142, Saudi Arabia; asalawi@jazanu.edu.sa (A.S.); yalmoshari@jazanu.edu.sa (Y.A.); malshamrani@jazanu.edu.sa (M.A.); 2 Faculty of Pharmacy, University of Central Punjab, Lahore 54000, Pakistan; tehseen.riaz@ucp.edu.pk (T.R.); hafsaihsan@ucp.edu.pk (H.I.); hmdbut@ucp.edu.pk (M.H.B.); rahima.khan888@gmail.com (R.K.); imtiaz.majeed@ucp.edu.pk (I.M.); 3 Department of Pharmacy, Hazara University, Mansehra 21120, Pakistan; waqar.aman@ucp.edu.pk

**Keywords:** hydrogel, free radical polymerization, β-CD, favipiravir

## Abstract

Nowadays, the use of statistical approaches, i.e., Box–Bhenken designs, are becoming very effective for developing and optimizing pharmaceutical drug formulations. In the current work, a Box–Bhenken design was employed using Design Expert version 11 to develop, evaluate, and optimize a hydrogel-based formulation for sustained release of an antiviral drug, i.e., favipiravir. The hydrogels were prepared using the free radical polymerization technique. β-Cyclodextrin (β-CD), *N*,*N*′-methylenebisacrylamide (MBA), acrylic acid (AA), and potassium per sulfate (KPS) were used as oligomer, crosslinker, monomer, and initiator, respectively. Three variables, including β-CD (X_1_), MBA (X_2_), and AA (X_3_) were used at various concentrations for the preparation of hydrogels, followed by evaluation of a sol–gel fraction, swelling, porosity, chemical compatibilities, in vitro drug release, and entrapment efficiency. The results of the studies revealed that the degree of swelling was pH dependent, the best swelling being at pH 7.2 (1976%). On the other hand, for the low sol fraction of 0.2%, the reasonable porosity made the hydrogel capable of loading 99% favipiravir, despite its hydrophobic nature. The maximum entrapment efficiency (99%) was observed in optimized hydrogel formulation (F15). Similarly, in vitro drug release studies showed that the prepared hydrogels exhibited a good, sustained release effect till the 24th hour. The kinetic modelling of drug release data revealed that the Korsmeyer–Peppas model was best fit model, describing a diffusion type of drug release from the prepared hydrogels. Conclusively, the outcomes predict that the hydrogel-based system could be a good choice for developing a sustained-release, once-daily dosage form of favipiravir for improved patient compliance.

## 1. Introduction

Innovative designs and breakthroughs in biomedical research aid in the development of better drug delivery systems. Oral administration is one of the most comfortable administration routes, and it also reduces costs and enhances patient compliance. In oral administration, the main challenge is to safely transfer the drug into the intestine; many orally taken drugs are absorbed via the gastrointestinal tract (GI). Furthermore, other difficulties are also faced, such as poor permeability throughout the GI tract, proteolytic degradation of the drug, and acid catalysis [1]. To conquer these difficulties for effectual delivery, pharmaceutical science requires innovative techniques of drug delivery, which also include protection of the drug through a hydrogel network [2].

A hydrogel is a three dimensional network of synthetic and natural polymers, and it has the capacity to absorb an enormous amount of biological fluid and water [3]. Hydrogels have been used in a variety of applications, including tissue engineering, cell therapy, and drug delivery, due to their broad potential to hold therapeutic chemicals in a depot-like structure that allows for sustained release delivery [4]. Due to their soft consistency, high water content, and porosity, they thoroughly simulate natural biomaterial more than any other synthetic biomaterials [5].

β-CD is an oligosaccharide that contains an inner hydrophobic cavity and an outer hydrophilic shell. They have the distinct ability to enhance the physiochemical properties of drugs through complexation with water-soluble drugs [6,7]. They enhance the stability, bioavailability, and solubility of a drug in hydrogel form [8]. In a hydrogel, β-CD can be added to form physically or chemically crosslinked networks for drug delivery [9]. 

In the production of a hydrogel, a monomer, such as acrylic acid, can also contribute effectively, and has been used extensively for their preparation. Comparatively, it (AA) has excellent water absorption capacity and acts as a multicomponent or single system [10]. One benefit of AA is that a change in polymer composition leads to different drug release properties depending on the pH of the environment. It is very much important to select a suitable crosslinker, such as *N*-*N*′-methylenebisacrylamide (MBA), to crosslink the polymer with the monomer to prepare a hydrogel with the desired characteristics, using the free radical polymerization method [11]. Meanwhile, a good initiator should also be the part of this system to initialize the free radical polymerization. Potassium persulfate (KPS) is one of the more effective and commonly used initiators in the free radical polymerization technique [12]. It is a white, crystalline, non-inflammable salt that is easily soluble in water as well [13].

Favipiravir is an antiviral board-spectrum drug that was approved by Japan for influenza virus treatment and an analog of purine nucleic acid [14]. It is capable of potently and selectively preventing RNA polymerase (RNA dependent) from translating influenza’s RNA, and that of various different RNA viruses [15]. 

In the current study, said drug was loaded in a sustained-release hydrogel formulation prepared via free radical polymerization technique, and optimized by Design Expert software. Design Expert is not only helpful for designing and evaluating a formulation, but it is also useful for optimizing a formulation. Hence, we tried to use the said software to avoid the tedious trial and error method and to preserve the useful material. 

## 2. Materials and Methods

Favipiravir was gifted by CCL Pharma (Lahore, Pakistan). The oligomer β-cyclodextrin (≥97%), potassium per sulfate (KPS) (≥99%) and *N*-*N*′-methylene bis acrylamide (MBA) (99%) were acquired from Sigma Aldrich, St. Louis, MO, USA. Distilled water and other excipients were obtained from the Post Graduate Research Laboratory, Faculty of Pharmacy, University of Central Punjab, Lahore, Pakistan.

### 2.1. Preparation of Hydrogel Formulations

The hydrogels were prepared using Design Expert software (Version 13, Stat-Ease Inc., Minneapolis, MN, USA), followed by employing the Box–Behnken approach to design the trials. In these trials, β-CD, A.A, and MBA were variable, but the quantity of initiator was kept constant (Table 1).

**Figure 1:**
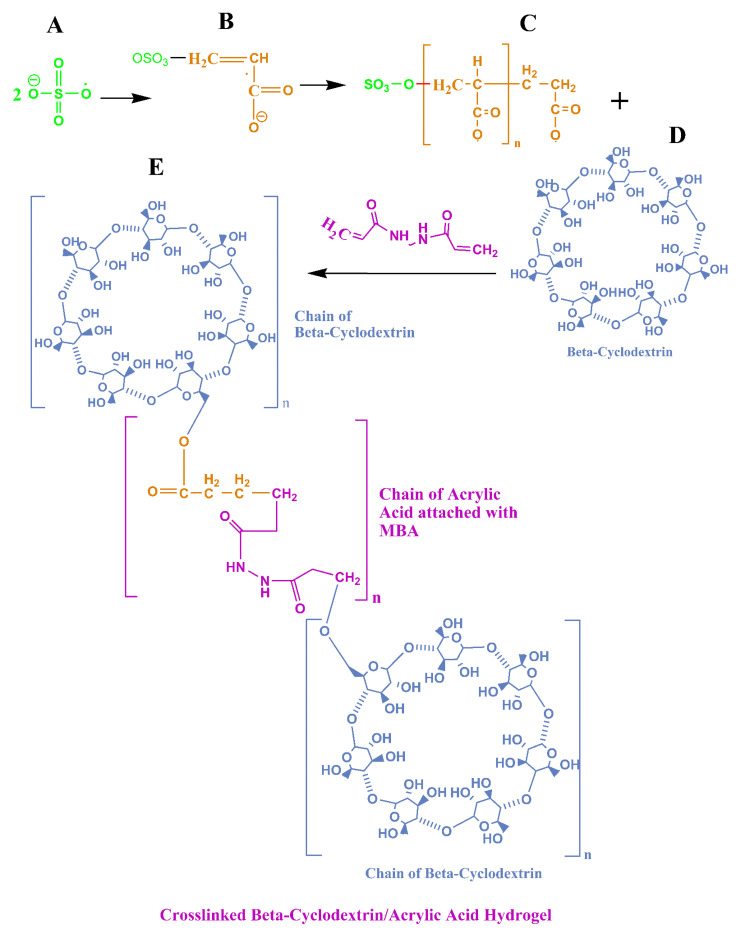
Describing the mechanism of free radical polymerization: how the heating helped the KPS to form free radicals (**A**), followed by its attachment to the acrylic acid to form monomer radicals (**B**). Afterward, a macro-radical was formed by attachment of monomers in the form of a chain (**C**). In the next step, this macro-radical crosslinked with the beta-cyclodextrin, in the presence of MBA (**D**), resulting in the formation of a crosslinked β-CD/AA hydrogel network (**E**).

Initially, the required amount of β-CD was dissolved in a suitable volume of distilled water under continuous stirring, using a hot plate magnetic stirrer at 40 °C. After that, the monomer was poured in a separate beaker, followed by the addition of a fixed amount of initiator (KPS) in it under continuous stirring. The mixture of monomer and initiator was added to a β-CD solution by continuing to stir with a magnetic stirrer. Finally, MBA (crosslinker) was added to this mixture with stirring, and the final volume was made up with water to 10 mL. The final mixture was transferred to glass test tubes and then placed in a water bath at 70 °C. The hydrogels settled in different time intervals depending on the concentration of crosslinker (Figure 1). After that, the congealed hydrogels were cooled at room temperature and cut into cylindrical discs, each 2 mm in thickness. The discs were washed with a mixture of ethanol–water (30:70) to remove unreacted catalyst and monomer. The washed hydrogels were dried in an oven at 50 °C for 2 to 3 days. When the hydrogel discs were completely dried, they were removed from the oven [16].

### 2.2. Drug Loading

For drug loading, a 1% favipiravir solution was prepared by dissolving favipiravir in ethanol using a hot plate magnetic stirrer, and the pH was adjusted to 7.2 by adding triethanolamine. Triethanolamine acts as a buffer solution and is used to adjust the pHs of hydrogels [17]. A 2 mm sized disc of dried hydrogel (0.32 ± 0.08 g) was immersed in a solution of favipiravir at 25 °C, until a constant weight was achieved. The drug-loaded hydrogel discs were taken out from the solutions of drug and washed with distilled water to remove any residual drug mass, followed by drying in the oven for 2 to 3 days at 40 °C.

### 2.3. Numerical Optimization

Numerical optimization was performed using different studied responses, including the degree of swelling, gel fraction, in vitro drug release, entrapment efficiency, and porosity of both hydrogel formulations. These all responses were added into the Design Expert software, and ANOVA was employed to correlate the experimental consequences of all trail formulations for the prepared hydrogels. These outcomes were than used to find out the optimized formulation of hydrogel having the desired characteristics.

For the numerical optimization of β-CD hydrogel, standards were set by defining amounts of monomer, polymer, and crosslinker. In vitro drug release and porosity were set to the targets of 100% and 200% respectively. The gel fraction (99.8) and the entrapment efficiency (99.69) were set to their maximum levels. The swelling of the hydrogel was targeted at pH 7.2. The resultant optimized formulation having 0.906 desirability had a composition of 0.500 g of β-CD, 8 g of acrylic acid, and 0.049 g of MBA. 

### 2.4. Characterization of Hydrogels

Hydrogels were characterized by various parameters.

#### 2.4.1. Organoleptic Evaluation

The prepared hydrogels were visually observed for color, shape, and homogeneity. For the detection of surface homogeneities and other abnormalities, such as discoloration, aggregation, or spotting, the hydrogels were visually examined.

#### 2.4.2. Swelling Studies

The swelling ratio of each hydrogel was studied using buffer solutions at different pHs: 1.2, 6.8, and 7.2. Dried hydrogel discs were dipped in buffer solutions until a constant weight was achieved. At a predetermined interval of time, the swollen hydrogel was taken out of the buffer solution, and using filter paper, excess liquid was dried, followed by weighing. This process was continued for up to 72 h. The hydrogel swelling ratio was calculated by using the equation;

(1) Swelling ratio=(Ws − Wd)/Wd ×100

In Equation (1), Ws represents the swollen hydrogel weight and Wd represents the weight of dried hydrogel [18]. 

#### 2.4.3. Sol–Gel Fraction

The dried hydrogel discs were soaked in distilled water at 37 °C for up to 48 h. Swollen hydrogel discs were dried in an oven for up to 50 °C until a constant weight was achieved and then weighed. The sol fraction and gel fraction were calculated by using the equation:

(2) Sol fraction (%)=[Wo− Wi/ Wo]×100 

(3) Gel fraction (%)=100−Sol fraction

In Equation (2), W_o_ represents the weight of the hydrogel before extraction and W_i_ represents the weight of the hydrogel after extraction [19]. 

#### 2.4.4. Porosity Measurement

For the determination of porosity, the solvent replacement method was used. In this method, the dried hydrogel was immersed in ethanol, for 24 h. After 24 h, the hydrogel was blotted to remove excess ethanol and then weighed. The hydrogel porosity was calculated using the formula [20]:

(4) Porosity =(M2 M1) ρV ×100

In Equation (4), M1 is hydrogel mass before dipping in ethanol, M2 is the hydrogel mass after dipping in ethanol, ρ represents the absolute ethanol density, and the hydrogel volume is denoted by V. 

#### 2.4.5. In Vitro Drug Release Studies

Dissolution studies of formulation (drug loaded) were carried out by using dissolution apparatus USP II (paddle apparatus) at pH 7.2 and 1.2. Phosphate buffer at pH 7.2 and pH 1.2 were used as media. Temperature was maintained at 37 °C  ±  0.2, and speed of the paddle was 50 rpm. Five milliliters of sample were extracted at defined intervals of time and replaced with fresh medium to maintain a constant volume of dissolution medium. By using the UV/vis spectrophotometer, the drug released was calculated by determining the absorbance at 233 nm. The same procedure was performed for every formulation to study the in vitro drug release [21].

(5) % Drug Release = Amount of drug present in sample/Amount of drug added ×100

#### 2.4.6. Drug Entrapment Efficiency

The dried hydrogel containing favipiravir was added to ethanol (50 mL) for 24 h to swell. The swollen hydrogel was the removed from ethanol, crushed using a mortar and pestle, and again added to the ethanol solution. For the formation of a homogenous solution, it was homogenized for 2 min at 13,000 rpm. The 50 mL of ethanol was added in this solution for the precipitation polymer mixture and then centrifuged for 5 min, followed by filtration. The amount of favipiravir was investigated using a UV spectrophotometer with a wavelength of 233 nm. The amount of favipiravir was determined by making serial dilutions of drug and then measuring absorbance using a UV spectrophotometer. The efficiency of drug entrapment was calculated using this formula [22].

Entrapment Efficiency (%) = Total amount of drug recovered/Total amount of drug added × 100(6)

#### 2.4.7. Drug Release Kinetics

The drug release kinetics of the favipiravir were studied by fitting the drug release data into zero-order kinetics, first-order kinetics, Higuchi’s equation, and the Hixson–Crowell model; and for better characterization of drug release mechanisms, the Korsmeyer–Peppas model was also applied. The release kinetics were evaluated using DD Solver software, and the R_2_ values were determined for each model to determine the drug release patterns of the formulations.

Zero-Order Equation

(7) Qt=ko t 

In Equation (7), Qt represents the percentage of drug released at time t, and ko is the release rate constant.

First Order Equation:

(8) 1n (100−Qt)=1n100−k1t 

In Equation (8), k1 is the release rate constant for the first-order kinetics.

Higuchi ’s Equation:

(9) Qt=kH t^(1/2)

In Equation (9), kH represented the Higuchi release rate constant.

Hixson-Crowell Model:

(10) (100−Qt)^(1/2)=〖100〗^(1/3)−kHC t

In Equation (10), kHC was the Hixson-Crowell rate constant.

Korsmeyer–Peppas Model:

(11) Qt/Qe= KKP t^n

In Equation (11), Qt/Qe is the fraction of the drug released at time t, KKP and n are the constants corresponding to the structural and geometric characteristics of the device, and the release exponent is indicative of the mechanism of the drug release [23]. 

#### 2.4.8. FTIR Analysis

FTIR spectra of AA, MBA, KPS, β CD, favipiravir, drug-loaded disc, and drug-unloaded disc of hydrogel were acquired. Hydrogel samples were examined using attenuated total reflectance (ATR-FTIR), Agilent, Santa Clara, CA, USA in the range of 4000–600 cm^−1^. It was used for the identification and quantification of functional groups.

#### 2.4.9. X-Ray Diffraction

The X-ray diffraction of the pure drug favipiravir and the drug-loaded hydrogel was conducted using an X-ray diffractometer (JDX-3532, JEOL, Akishima, Japan) at 40 kV, and the scanning range was 10 °C to 45 °C. The XRD was performed to check the nature of the components, i.e., amorphous and crystalline [24].

#### 2.4.10. Scanning Electron Microscopy (SEM)

Scanning electron microscopy (SEM) with EDX and an E-beam Lithograph FEI Nova 450 NanoSEM (Thermo fisher scientific, Waltham, MA, USA) machine was used. SEM was conducted to study the size and morphology of each hydrogel formulation. The hydrogel surface was analyzed using the SEM at different magnification levels [25]. 

## 3. Results and Discussions

### 3.1. Swelling Studies

Swelling of β-CD hydrogels was determined at pH 6.8, pH 1.2, and pH 7.2. Swelling studies were performed on all trial β-CD hydrogels. The quantity of KPS was kept constant in all formulations, but the concentrations of oligomers, crosslinkers, and monomers varied among all formulations. The effects of varying concentrations of monomer, crosslinker, and oligomer on swelling behavior were studied. 

**Figure 2:**
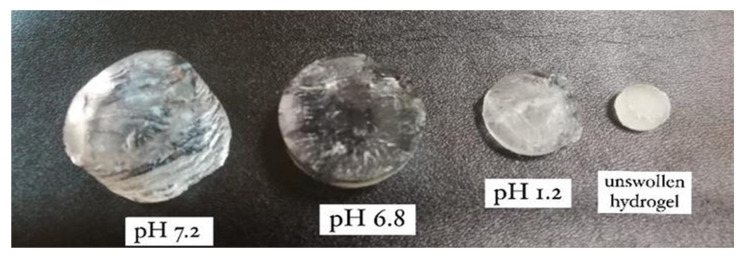
Illustrating the pH-dependent swelling behavior of β-CD hydrogels: greater swelling at basic pH (pH 7.2) compared to pH 6.8 and pH 1.2.

β-CD-based hydrogels showed more swelling at pH 7.2 compared to pH 1.2 (Figure 2). This behavior occurred due to the presence of carboxyl groups in the hydrogel network, and ultimately, the swelling index increased at a higher pH compared to a lower pH. At alkaline pH (7.2), these hydrogels swell due to intra-ionic repulsion between the protonated carboxyl groups. In the polymeric network, carboxylate anions have a stronger tendency to solvation as compared to non-ionic groups in alkaline and aqueous media [16].

#### 3.1.1. Effect of Crosslinker (MBA) on Hydrogel Swelling 

The concentration of the crosslinking agent is an important parameter in the preparation of a hydrogel because it directly affects the swelling. In the current study, results depict that, with the increase in crosslinker concentration, the swelling index of the hydrogel ultimately decreased. However, Ninciuleanu et al. reported that with the increase in MBA concentration (crosslinker), the swelling degree of the hydrogel increased due to the higher crosslinking density [26]. The water absorption capacity of the hydrogel was lower as a result of more crosslinking agent. The phenomenon of water diffusion into the network of a hydrogel can be explained easily, but with a higher crosslinking density, it may be difficult. This is because of a higher density of crosslinking agent and smaller mesh size.

#### 3.1.2. Effect of Monomer on Hydrogel Swelling 

The effect of acrylic acid (monomer) composition on the swelling index of the hydrogels showed that by increasing its concentration, the swelling degree of the hydrogels was increased. Sindhu et al. reported that with the increase in monomer concentration, swelling of hydrogels increased due to ionization of the carboxylic group at higher pH levels [27]. Additionally, the presence of acrylic acid in a hydrogel makes it more ionic, which leads to increase in hydrogen ions concentration. 

### 3.2. Sol–Gel Fraction

This gel fractionation was performed to calculate the quantity of un-crosslinked polymer that remained in the hydrogel. The β-CD hydrogel had a gel fraction range from 82.13 to 99.8%. In the current study, we observed that as the concentrations of monomer, polymer, and crosslinker increased, the gel fraction percentage in the hydrogel increased. Similar results were reported by Barkat, K et al.: increases in concentrations of crosslinker, monomer, and polymer resulted in improved gel fraction% due to the presence of primary radicals (active) in the monomers [28]. 

### 3.3. Porosity

This test was performed to check the porous structure of each hydrogel. The β-CD hydrogels had porosity ranging from 81.13 to 256.7%. The increased quantity of monomer resulted in an upsurge of porosity percentage, but different phenomena were observed if we increased the MBA concentration. Similar results were reported by Shabir et al. in LSH-co-MAA based formulations. They reported that with the increase in monomer concentration, the viscosity of the solution raised and stopped the bubbles escaping from the solution, thereby forming interconnected channels that increased the porosity. Porosity was decreased as MBA concentration raised [29].

### 3.4. Drug Entrapment Efficiency

The drug entrapment efficiencies of β-CD hydrogels of F2 and F13 were found to be 97.425% and 96.025%, respectively, and the overall range of drug entrapment efficacy was 79.475 to 99.275% (Table 2). The results show that with the increases in amounts of polymer and monomer, drug entrapment efficiency eventually increased. These results are supported by a study conducted by Nautiyal, U. et al. They reported that monomer and polymer increased the drug entrapment efficiency [22]. 

The drug entrapment efficiency of the hydrogels increased with the amount of crosslinker. High crosslinking density can lead to decreased elasticity of polymeric structure and might decrease the drug entrapment. These results are comparable to those of a study reported by Malik, N. S. et al., where they formulated hydrogel based on CS/XG [30].

### 3.5. In Vitro Drug Release Studies 

**Figure 3:**
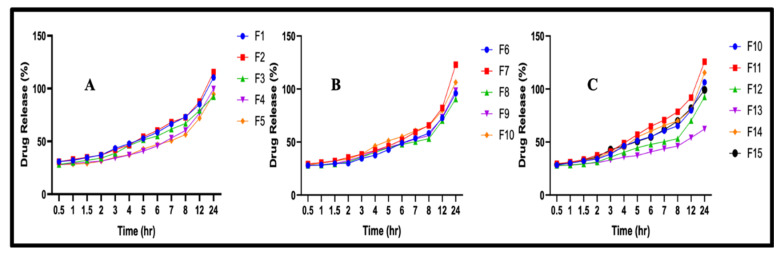
The graphs show in vitro drug release of favipiravir from different formulations of β-CD hydrogels: (**A**) F1–F5; (**B**) F6–F10; (**C**) F11–F15. They describe the sustained release behavior.

Using the USP dissolution apparatus, we calculated the drug release of drug-loaded discs of both types of hydrogel over 24 h. The in vitro drug release of β-CD hydrogels had a range from 62.32 to 125.2% (Figure 3). A study by Ramadan et al. showed the dependence of hydrogels on the concentrations of polymer, monomer, and crosslinker [31]. 

#### 3.5.1. Effect of Concentration of β-CD on In Vitro Drug Release

According to the findings, the in vitro drug release increased with the concentration of β-CD. β-CD creates a larger hydrogel network and allows more drug to escape in the gel and also in its surrounding medium. These findings are consistent with Dong et al., who created dual-crosslinked hyaluronic-acid hydrogels. They reported an increase in drug release rate with an increase in polymer concentration [32].

#### 3.5.2. Effects of Monomer and Crosslinker Concentrations on In Vitro Drug Release

The amount of monomer increased the drug release from the hydrogel, but crosslinker played a different role and decreased the drug release. The study reported by Bueno et al. reported similar phenomena in GG/PVP copolyacrylic-acid-based hydrogels. They discovered that with the increase in acrylic acid concentration, the drug release rate also increased. However, with the increase in crosslinker concentration, the penetration of the fluid decreased due to interconnections between monomer and polymer [33]. 

### 3.6. Drug Release Kinetics 

The drug release kinetics and mechanisms of hydrogels were observed at pH 7.2 by applying various kinetic models through DD Solver software, and acquiring their correlation coefficient values (R^2^). The kinetic results showed that the hydrogels have Fickian diffusion, and detailed results are in Table 3.

The R^2^ values for the Korsmeyer–Peppas model dominated for β-CD hydrogels over all other applied kinetic models. The R^2^ and values of first order kinetics suggest that the release of the drug would be dependent upon the initial concentration of the drug in the hydrogel. They also indicate that the formulations might have sustained release but not controlled release. In a different study, Sarfraz et al. prepared β-CD-based hydrogels and described comparable findings [8]. The value of “n” in all β-CD formulations was as less than 0.5, which confirmed Fickian diffusion.

### 3.7. Mathematical Modeling

Mathematical modeling was performed to find the presence of any uncertainties in the observed data. This was performed to characterize the chosen model and assess its ability for effectively evaluating the formulated hydrogels. For this purpose, the quadratic model was chosen, and mathematical modeling was performed by using the Design Expert software and calculating the responses and variables in mathematical expressions.

Polynomial equation:

(12) $$Y=X1-X2+X3+X1X2+X1X3-X2X3+X1^{2}-X2^{2}+X2^{2}$$

#### 3.7.1. Response 1: Degree of Swelling

**Figure 4:**
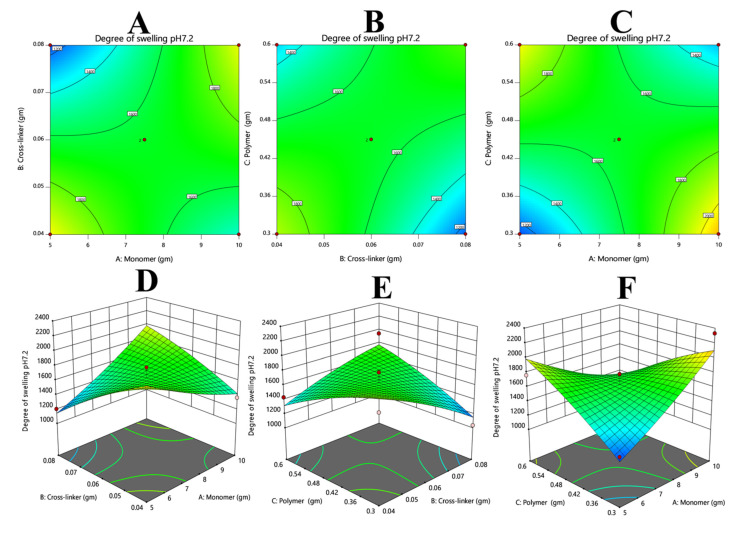
Illustrates the effects of varying concentrations of monomer and crosslinker (**A**,**D**), polymer and crosslinker (**B**,**E**), and polymer and monomer (**C**,**F**) on the degree of swelling. It is clearly evident that both polymer and monomer had a positive effect on the swelling index of a hydrogel, whereas the crosslinker showed the opposite effect.

The swelling results were added via Equation (12). From the polynomial equation, and contour and 3D graphs, it can be observed that the overall response was constructive. The positive values of X1 and X3 suggest that both variables will improve the swelling. On the other hand, the negative value of X2 illustrates that by increasing the value of X2, we can decrease swelling (Figure 4).

(13) Swelling at pH 7.2=+64.87−73.25+9.37+353.75−418.50+302.25+44.50−47.25−56.50

#### 3.7.2. Response 2: Gel Fraction

**Figure 5:**
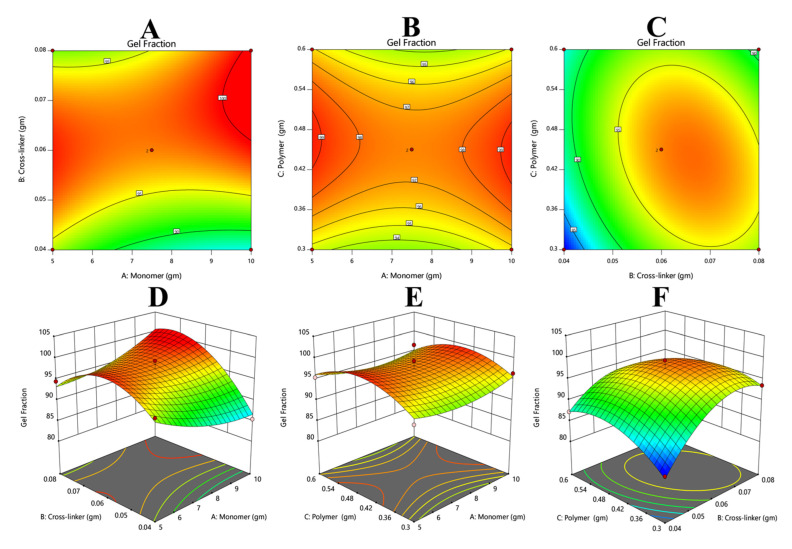
Describing the effects of varying the concentrations of crosslinker and monomer (**A**,**D**), polymer and monomer (**B**,**E**), and polymer and crosslinker (**C**,**F**). The pictorial representation describes that increasing the concentration of crosslinker could be the cause of an increase in gel fraction and vice versa.

The results of gel fraction were added via Equation (12). From the polynomial equation and contour and 3D graphs it could be observed that the overall response is constructive (Figure 5). The positive values of X1, X2, and X3 suggest that these variables will increase the gel fractions of hydrogels.

(14) Gel Fraction%=+0.0462+3.36+0.2875+3.63−0.5325−2.54+1.82−5.75−3.95

#### 3.7.3. Response 3: Porosity

**Figure 6:**
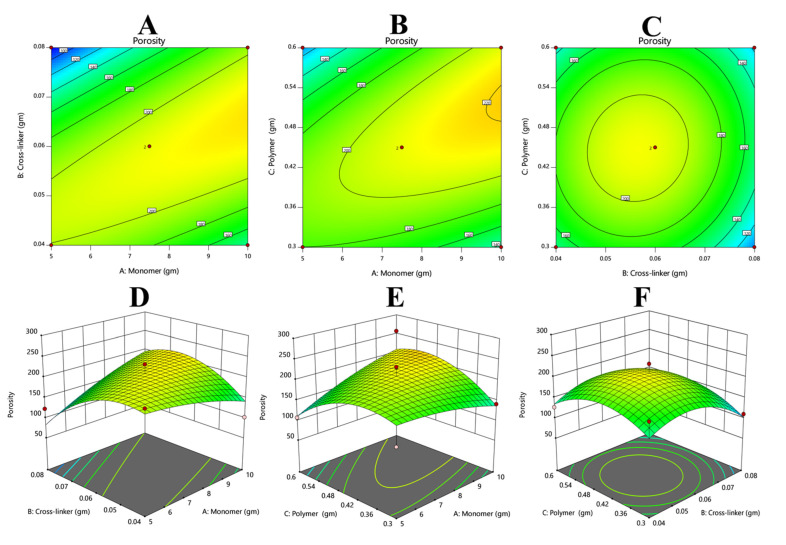
Illustrating that the increase in the concentration of crosslinker might be the cause of decreased porosity of the hydrogels and vice versa. (**A**,**D**) Describes the comparative effects of crosslinker and monomer, (**B**,**E**) the comparative effects of polymer and monomer, and (**C**,**F**) the comparative effects of polymer and crosslinker on the porosity of the hydrogels.

The results of porosity were added via Equation (12). From the polynomial equation and contour and 3D graph, it could be observed that the overall response was constructive. The positive values of X1 and X3 suggest that both variables will enhance the porosity. However, the negative value of X2 depicts that by increasing the value of X2, porosity decreased (Figure 6).

(15) Porosity%=+13.84−15.63+0.7125+42.50+35.17+6.75−10.59−44.51+40.34

#### 3.7.4. Response 4: Entrapment Efficiency

**Figure 7:**
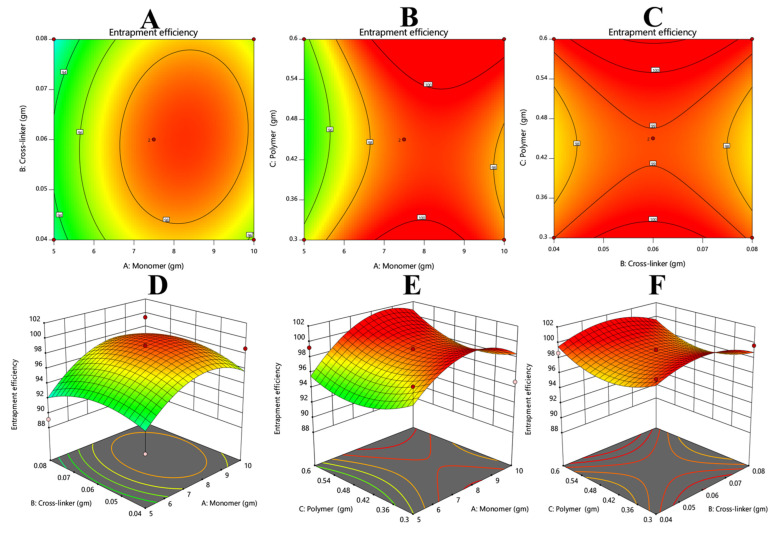
Describing the relationship between entrapment efficacy and the variable concentrations of crosslinker, monomer, and polymer. (**A**,**D**), (**B**,**E**), and (**C**,**F**) describe that the drug entrapment efficiency would be improved with increases in concentrations of monomer and polymer, but an increase in the concentration of crosslinker could be the cause of a decrease in the drug entrapment.

The results of the entrapment efficiency were added via Equation (12). From the polynomial equation and contour and 3D graph, it could be observed that the overall response was constructive. The positive value of X1 suggests that this variable will enhance the EE %. However, the negative values of X2 and X3 show that by increasing the values of X2 and X3, drug entrapment efficiency decreased (Figure 7).

(16) EE%=+1.77−0.0338−0.3250+0.4275+0.8550+0.0700−2.96−1.63+1.90

#### 3.7.5. Response 5: In Vitro Drug Release

**Figure 8:**
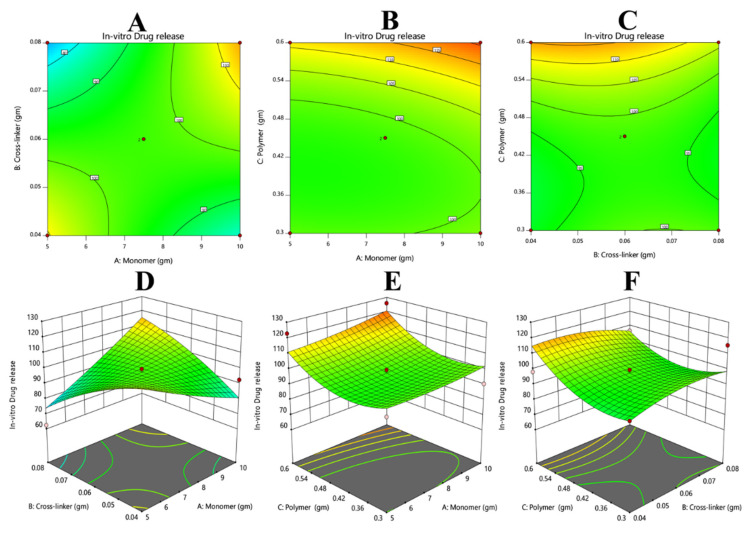
(**A**,**D**) Shows the crosslinker has a more pronounce effect on decreasing the release of the drug as compared to the monomer; (**B**,**E**) shows that the monomer was more effective at controlling the drug release as compared to the polymer. (**C**,**F**) The crosslinker was more efficient at reducing the release of the drug as compared to the polymer.

The results of in vitro drug release were added via Equation (12). From the polynomial equation and contour and 3D graph, it could be observed that the overall response was constructive. The positive values of X1 and X3 suggest that these variables can enhance the drug release, and the negative value of X2 illustrates that it will decrease the drug release (Figure 8).

(17) Drug release=+3.00−0.5500+7.54+17.76+1.79−3.80+1.39−3.95+9.27

### 3.8. Numerical Optimization

The results of optimized formulations of β-CD hydrogels were compared with the predicted outcomes obtained from Design Expert software. The results of optimized hydrogel showed no significant differences from predicted outcomes, and the optimized formulation was considered successful, having all the required characteristics, such as optimal porosity, gel fraction, degree of swelling, entrapment efficiency, and in vitro drug release. Table 4 shows a comparison of the predicted results and the results obtained from optimized formulations.

### 3.9. Fourier Transform Infrared Spectroscopy (FTIR)

**Figure 9:**
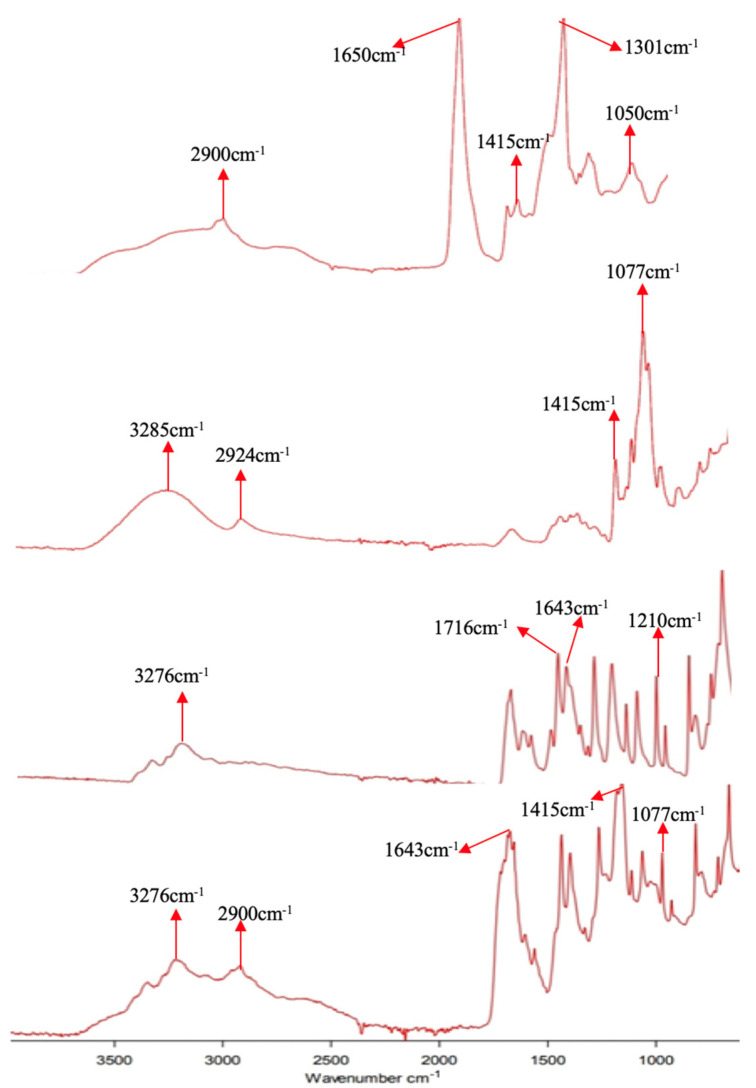
The FTIR spectra of (A) β-CD hydrogel (unloaded hydrogel) reviled the emergence of a new peak at 1052 cm^−1^ that confirmed the development of the hydrogel network by the formation of a link between the OH of β-CD and the COOH group of acrylic acid. B describes the FTIR spectrum of β-CD, C shows the spectrum of pure drug (favipiravir), and D illustrates the spectrum of drug-loaded β-CD hydrogel. The presence of favipiravir’s characteristic peaks in the drug-loaded hydrogel confirmed the compatibility of the drug with excipients.

The FTIR spectra helped to indicate the functional groups of active constituents found by the peak values of the IR spectrum. FTIR spectra of unloaded hydrogel, favipiravir, β-CD, and drug-loaded hydrogel are presented in Figure 9.

The FTIR spectrum of the pure favipiravir drug showed peaks at various points. One peak of –OH stretching was observed at 3276 cm^−1^. The strongest peak was observed at 1716 cm^−1^ corresponded to C=O stretching. The C=N stretching peak was observed at 1643 cm^−1^. A band at 1210 cm^−1^ is owed to C–F stretching [34]. In the spectrum of β-CD, peaks of C–O–C and C–O stretching vibration are shown at 1077 and 1415 cm^−1^, respectively. The –CH stretching vibration’s peak was found at 2924 cm^−1^. The peak at 3285 cm^−1^ is of –OH stretching [35]. An FTIR scan of the unloaded hydrogel shows different peaks related to the materials used in the formulation of hydrogel, such as at 2900 and 1050 cm^−1^, which might be indicating the presence of –CH stretching and C–O stretching, respectively. 

The drug and excipients used in the preparation of hydrogel were compatible with each other, as the characteristic peaks of drug (3276 cm^−1^, 1643 cm^−1^, etc.) are clearly visible in the drug-loaded hydrogel.

A comparison of the pure β-CD and the β-CD hydrogel spectra revealed the emergence of a new C–O–C bond peak at 1052 cm^−1^, which confirmed the polymerization leading towards the development of the hydrogel network. The link was established between the –OH group of β-CD and the –COOH group of acrylic acid. This validated the successful crosslinking for the preparation of the hydrogel. This is also described in Figure 1.

### 3.10. X-Ray Diffraction (XRD)

**Figure 10:**
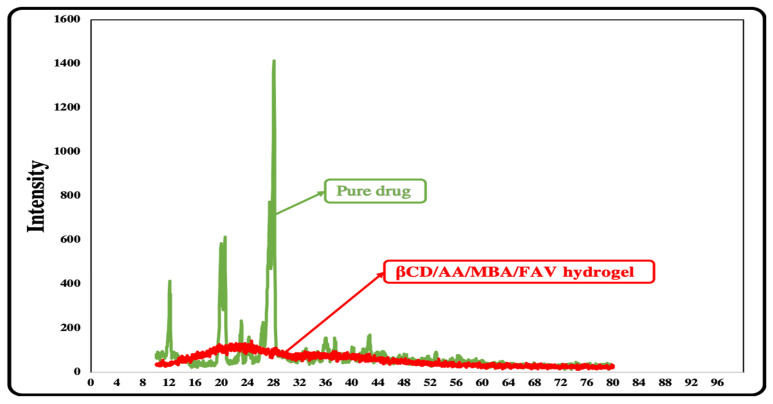
Illustrating XRD diffraction of pure drug (favipiravir) and βCD/AA/MBA/FAV hydrogel. The XRD of the pure drug shows sharp and intense peaks, indicating crystalline structure, but the XRD of the drug-loaded hydrogel shows no sharp peaks and crystalline structure, which indicates that crystallinity of the drug is masked in nanocomposite hydrogel form.

The diffraction patterns of pure drug (favipiravir) and βCD drug-loaded hydrogels were compared. The sharp and intense peak of the favipiravir drug was recorded at a 28° angle, which showed that the drug was crystalline in nature. Diffused peaks were recorded in the diffractogram of the drug-loaded hydrogel of βCD, rather than sharper peaks. These diffused peaks showed that the drug was present in amorphous form in the drug-loaded hydrogel formulation. The amorphous form indicated that drug was successfully loaded into the βCD hydrogel. The βCD has a basic crystalline structure that was changed, and high crystalline peaks and high intensity were exchanged with decreased and weak-intensity peaks, indicating effective polymer grafting (Figure 10).

### 3.11. Scanning Electron Microscopy (SEM)

**Figure 11:**
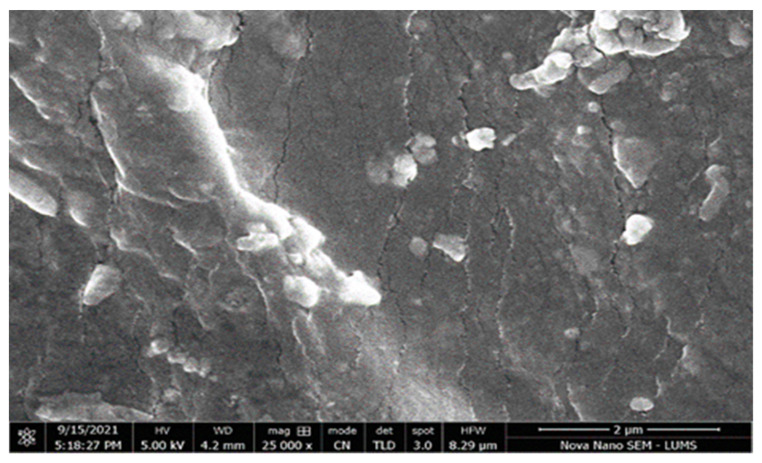
SEM image of βCD/AA/MBA/FAV hydrogel at 25,000 magnifications: highly porous, compact, and rough surface.

SEM of the β-CD/AA/MBA/FAV hydrogel was conducted at different resolution levels to evaluate the surface morphology of the hydrogels. The βCD drug-loaded hydrogel SEM image showed that the hydrogel’s structure was highly porous, compact, and had a rough surface. Ahmed et al. observed similar results. They developed a βCD-based hydrogel to improve the solubility of acyclovir. In Figure 11, big cracks and wrinkles are shown, which could have been due to the gel partially collapsing during dehydration. At a low pH due to protonation of the hydrogel contents, the pore size remained the same; however, at high pH, the pore size increased due to the creation of repulsive forces among the identical functional groups of the hydrogel’s contents.

## 4. Conclusions

The objective of the studies was achieved successfully, as the favipiravir hydrogels have the ability to deliver the drug sustainably over a 24 h period. The application of a statistical approach seemed to be fruitful, as it not only helped in the design and evaluation, but also assisted in the optimization of the hydrogel formulation. The experimental work was found to be reproducible, since the findings of the optimized formulation were comparable to those predicted by Design Expert for the optimized formulation. Hence, it can be concluded that the hydrogel drug delivery system is effective at loading high doses of the drugs and for sustained release delivery of the loaded APIs. Furthermore, Design Expert will be a useful tool for time saving and cost effectiveness by reducing unnecessary trials for formulation optimization.

## Figures and Tables

**Table 1:** Ratio of excipients used in the preparation of β-CD hydrogels by Design Expert.

Serial Number	Monomer (Acrylic Acid) (G)	Crosslinker (MBA) (G)	Oligomer (β-CD) (G)
	X_1_	X_2_	X_3_
1	5	0.04	0.225
2	2.5	0.02	0.225
3	3.75	0.02	0.15
4	3.75	0.03	0.225
5	2.5	0.03	0.15
6	3.75	0.03	0.225
7	2.5	0.03	0.3
8	5	0.03	0.15
9	3.75	0.02	0.3
10	3.75	0.04	0.3
11	5	0.03	0.3
12	5	0.02	0.225
13	2.5	0.04	0.225
14	3.75	0.04	0.15

**Table 2:** Drug entrapment efficiency of prepared formulations.

Formulation	Absorbance	Total Drug Loaded (mg/mL)	Amount of Drug Recovered (mg/mL)	% Entrapment Efficiency
**F1**	1.645	5	3.97375	79.475
**F2**	2.004	5	4.87125	97.425
**F3**	1.766	5	4.27625	85.525
**F4**	1.786	5	4.32625	86.525
**F5**	1.656	5	4.00125	80.025
**F6**	1.65	5	3.98625	79.725
**F7**	1.712	5	4.14125	82.825
**F8**	1.756	5	4.25125	85.025
**F9**	1.765	5	4.27375	85.475
**F10**	1.732	5	4.19125	83.825
**F11**	1.656	5	4.00125	80.025
**F12**	1.702	5	4.11625	82.325
**F13**	1.976	5	4.80125	96.025
**F14**	1.876	5	4.55125	91.025
F15 *	2.041	5	4.96375	99.275

* Optimized formulation.

**Table 3:** R^2^ and coefficient values of Kinetic models used for β-CD hydrogels.

Formulation	Zero-Order		First Order		Higuchi Model		Korsmeyer-Peppas Model			Hixson-Crowell Model	
	R^2^	K_0_	R^2^	K_1_	R^2^	KH	R^2^	KKP	N	R^2^	KHC
F1	0.1431	6.296	0.8153	0.178	0.9415	24.318	0.9742	29.209	0.417	0.7717	0.049
F2	0.0057	6.481	0.8171	0.182	0.9546	24.874	0.9727	28.678	0.436	0.7854	0.051
F3	0.5570	5.593	0.7887	0.154	0.8823	21.967	0.9689	28.769	0.377	0.7162	0.043
F4	0.1417	5.450	0.7777	0.125	0.9276	20.713	0.9346	22.773	0.457	0.7477	0.036
F5	0.0333	5.224	0.7406	0.120	0.9272	20.007	0.9481	23.328	0.431	0.6852	0.034
F6	0.0008	5.315	0.7709	0.124	0.9346	20.350	0.9525	23.481	0.435	0.7213	0.035
F7	0.4206	6.333	0.7757	0.152	0.9509	23.673	0.9513	23.076	0.511	0.7618	0.042
F8	0.2832	5.095	0.6804	0.119	0.9079	19.700	0.9569	24.494	0.401	0.5960	0.034
F9	0.0223	5.405	0.7566	0.128	0.9397	20.701	0.9603	24.099	0.431	0.7033	0.036
F10	0.0278	5.939	0.8191	0.155	0.9565	22.819	0.9779	26.592	0.431	0.7736	0.043
F11	0.2447	6.916	0.8389	0.197	0.9776	26.279	0.9804	27.927	0.473	0.8440	0.061
F12	0.1924	5.148	0.6978	0.119	0.9175	19.837	0.9556	24.155	0.411	0.6225	0.034
F13	3.0774	3.929	0.8107	0.085	0.3238	15.913	0.9458	26.483	0.263	1.2802	0.024
F14	0.1024	6.321	0.8255	0.170	0.9646	24.148	0.9754	27.051	0.449	0.7885	0.047
OF	0.3685	5.844	0.8035	0.162	0.9127	22.781	0.9728	28.801	0.393	0.7437	0.045

**Table 4:** Comparison of the predicted outcomes and obtained results of β-CD hydrogels.

Parameter	Predicted Outcomes	Obtained Results
Porosity	200.000%	187%
Gel Fraction	93.745%	92.8%
Entrapment Efficiency	99.033%	98.73%
Degree of Swelling pH 6.8	147.629%	1362%
Degree of swelling pH 7.2	1556.189%	1770%
In vitro drug release	99.998%	98.419%

## Data Availability

All the required data have been represented in the manuscript.
